# Investigating the relationship between early cardiovascular disease markers and loneliness in young adults

**DOI:** 10.1038/s41598-024-65039-8

**Published:** 2024-06-20

**Authors:** Shradha Vasan, Michelle H. Lim, Nina Eikelis, Elisabeth Lambert

**Affiliations:** 1 Iverson Health Innovation Research Institute, Swinburne University of Technology, Melbourne, Victoria 3122 Australia; 2 Department of Mental Health Services, St. Vincent’s Hospital Melbourne, Melbourne, Australia; 3 Prevention Research Collaboration, Sydney School of Public Health, Charles Perkins Centre, The University of Sydney, Sydney, Australia; 4 School of Health Sciences, Swinburne University of Technology, Melbourne, Australia

**Keywords:** Psychology, Human behaviour

## Abstract

Loneliness is recognised as a risk factor for cardiovascular disease development. However, it is unclear whether loneliness itself or other closely related mental health symptoms, such as depression and social anxiety, are associated with the development of cardiovascular disease. In the present study, we examined the relationship between loneliness and several early cardiovascular disease markers in young adults, after controlling for depression and social anxiety. Sixty-six young adults (18–35 years old, *M*_*age*_ = 22.70; 75.8% females) completed psychological questionnaires and took part in several physiological tests assessing cardiovascular health (e.g., vascular function). Results revealed higher loneliness was significantly associated with shorter pulse transit time (β = − 0.70, *p* = 0.002; shorter pulse transit time is a subclinical marker for arterial stiffness). Additionally, results show that while loneliness and depression were both related to vascular dysfunction in young adults, the underlining physiological mechanisms through which they affect vascular function may be different. Specifically, higher loneliness was associated with increased arterial stiffness, whereas depression was associated with increased endothelial dysfunction (β = − 0.43,* p* = 0.04). Our findings indicate that presence of loneliness and depression in young adults may be accompanied by early indicators of poor cardiovascular health, such as arterial stiffness and endothelial dysfunction. Results from the study further support the link between loneliness and cardiovascular disease development.

## Introduction

Loneliness has been identified as a social determinant of health and a major public health issue^1–3^. It is characterised as a subjective distressing feeling of disconnect from others, accompanied by a desire for either more, or more fulfilling social relationships^4^. Subsequently, it is more closely related to the quality of one’s social relationships, as opposed to the quantity of social connections^5,6^. Furthermore, it is also a relatively common experience^3,7,8^. Indeed, a 2018 survey found 51% of Australian adults felt lonely at least 1 day a week^9^. For the majority of people, feelings of loneliness are transient and part of an adaptive experience that motivates them to form new social connections and maintain existing ones^10^. However, when left unchecked, temporary feelings of loneliness can develop into problematic levels of loneliness and have a negative impact on health^7,11,12^.

Substantial evidence from cross-sectional and longitudinal studies have established the link between loneliness and higher incidence of physical health diseases, especially cardiovascular disease (CVD)^13–16^. CVDs are the global leading cause for mortality^17^ and loneliness has been identified as a risk factor for a number of CVDs, including coronary heart disease and stroke^13–16^. Studies in this area have primarily focused on older adults as they were believed to be the age group most vulnerable to the negative effects of loneliness, and most likely to present with related CVD issues^13,15^. However, more recent research shows loneliness to be equally pervasive in younger adults (< 35 years old)^18–21^. Primary and secondary CVD prevention (e.g., individual lifestyle changes, early diagnosis) have been identified as necessary steps to help relieve the disease burden on individuals and the healthcare system^22,23^. In order to better understand how loneliness may be related to the development of CVD, it may be prudent to focus on young adults and assess their risk of developing CVD by utilising early markers of CVD. By using this approach, we can facilitate early diagnosis and intervention, thereby promoting better health outcomes.

CVD typically develops later in life, however, progression to CVD can be detected decades before (i.e., in young adulthood) in forms of metabolic abnormalities, such as increased blood pressure, higher body mass index (BMI) as well as early impaired vascular function and autonomic nervous system function (referred to as autonomic function)^22,24^. Hence, to better understand the relationship between loneliness and CVD progression, in the present study, we focus on early indicators of poor cardiovascular health (CVH, the term ‘CVH indicator’ is also referred to as ‘CVD marker’ or ‘CVD indicator’ throughout the article) which have been shown to be accurate and consistent predictors of CVD^22,24–27^. Previous studies investigating the relationship between loneliness and some more common CVH indicators (i.e., blood pressure, BMI, and heart rate variability (HRV), ^25–27^) have reported mixed results. Some studies found a relationship between higher loneliness, increased blood pressure, higher BMI (obesity), and lower HRV^14,28–32^. Other studies reported no associations between indicators of poor CVH and loneliness^33–36^. Notably, very few of these studies focused on young adults^29,32^. To the best of our knowledge, no studies have examined the association between loneliness and specific early markers of CVD, such as vascular and autonomic function in young adults.

Loneliness has a multifaceted and possibly confounding relationship with social anxiety and depressive symptomology (referred here as depression and social anxiety respectively). Loneliness may be an antecedent to depression^30,37^ but the relationship between loneliness and social anxiety has been shown to be bi-directional^38,39^. While some studies have controlled for either depression or social anxiety when investigating the influence of loneliness on more common CVH indicators (e.g., blood pressure, HRV^14,29,31,34^) in older adults, they have reported mixed findings, thus necessitating further investigation. Moreover, there has been little to no research differentiating the influence of depression *and* social anxiety on the relationship between loneliness and certain early markers of CVD (e.g., vascular and autonomic function) in young adults. To fully understand the *unique* relationship between loneliness and CVH markers in young adults, it is imperative to consider the influence of highly correlated mental health symptoms such as depression and social anxiety.

The primary study aim was to investigate the relationship between loneliness and CVH indicators (i.e., vascular function, autonomic function, BMI, and blood pressure) in young adults, after accounting for social anxiety and depression. We also examine potential differences in the associations between loneliness, social anxiety, and depression, and various CVH indicators in young adults.

## Method

Ethical approval was granted by the Swinburne University Human Research Ethics Committee (SUHREC) and the study was conducted in accordance with the SUHREC guidelines. Sixty-six young adults (18–35 years old) were recruited via social media advertisements, word of mouth, online forums, and a research training program for first year Bachelor of Psychology students. We adopted a cross-sectional study design. Participation was voluntary, an explanatory statement was provided, and informed consent was obtained before participants could partake in the study. The Bachelor of Psychology students involved in this study received course credit in return for participation, whereas community participants received a monetary reimbursement.

**Figure 1:**
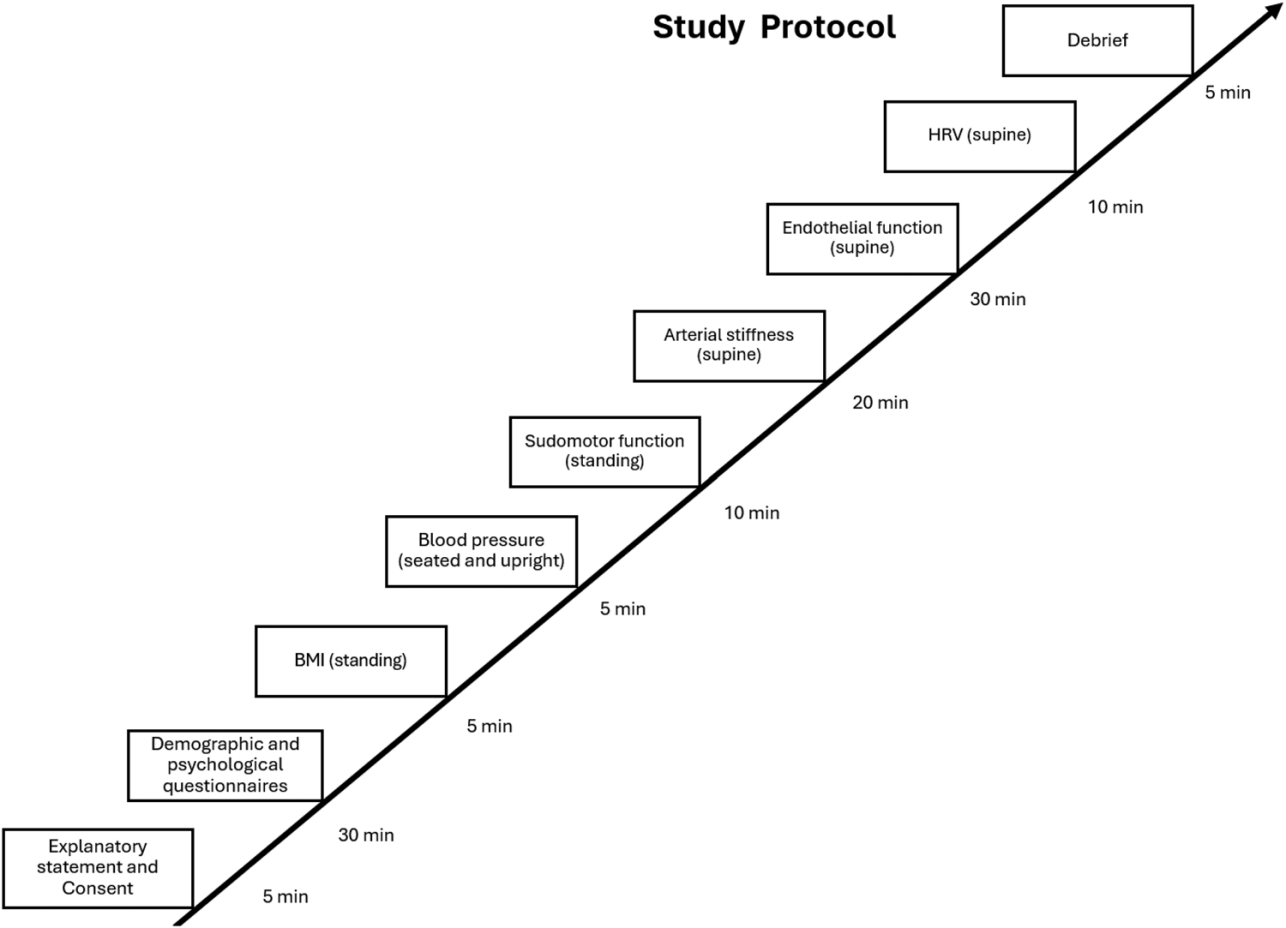
A visual representation of the laboratory session which illustrates the study protocol, the tests conducted, their duration, and the employed testing modality.

Study data was collected by a single investigator between September 2021 and July 2022. Respondents attended a two-hour laboratory session where after providing consent, they completed a short questionnaire which included demographic questions and the psychological measures. Next, participants height, weight, and BMI measurements were taken. After a 5-min rest, blood pressure measurements were taken while the participant was in an upright seated position (as per the guidelines of the National Heart Foundation of Australia). Specific details or steps for how the tests for each CVH/CVD indicator were carried out are included below, under each test description. Following blood pressure measurement, the participant completed the assessment for sudomotor function (measure for autonomic function), which took approximately 10 min to complete. Then, the participant was instructed to lie down on the clinic bed in a supine position, where they completed the test for arterial stiffness (measure for vascular function). This assessment took approximately 20 min to complete. Endothelial function (another measure for vascular function) was measured next. The test for this was 30 min long. Next, while in supine position, participants’ blood pressure, heart rate, and electrocardiogram were recorded for 10 min (measure for HRV). An illustration detailing the study protocol is included below in Fig. 1.

Demographic information was obtained, including age, gender, marital status, ethnicity, employment status, education level, living status, chronic CVD conditions (see Table 1).

### Psychological questionnaires

#### Loneliness

Loneliness was measured using the UCLA Loneliness Scale-Version 3 (UCLA-LS)^40^. The UCLA-LS includes 20 questions which assess feelings of loneliness over a one-month period. Responses are given on a 1 (*Never*) to 4 (*Always*) Likert-type scale. Composite scores are calculated by reverse coding the nine positively worded items and then adding the scores for all the questions. Score range is between 20 and 80, with higher score suggesting higher levels of loneliness. The UCLA-LS has been found to be a valid and reliable measure of loneliness across diverse populations (e.g., students, nurses, teachers, older adults^40^). Cronbach’s α for UCLA-LS in the present study was 0.95.

#### Social anxiety

Social anxiety symptoms were measured using the Social Interaction Anxiety Scale (SIAS)^41^. We used the straightforward version (S-SIAS) of the SIAS which consists of only the 17 negatively worded items, as opposed to the original SIAS, which includes 20 negative and positive items. S-SIAS is a more appropriate scale to use in the present study as the negatively worded statements have shown to be better indicators of social interaction anxiety, whereas the positively worded questions align more closely with constructs such as extraversion^42,43^. S-SIAS utilises a 0 (*Not at all characteristic of me*) to 4 (*Extremely characteristic of me*) Likert-type scale. The S-SIAS measure a person’s general levels of anxiety when initiating and maintain social interaction. Total scores are calculated by adding the individual scores for all 17 items. Score range is between 0 and 68, with higher scores indicating higher social interaction anxiety or increased social anxiety symptoms. SIAS has demonstrated excellent psychometric utility as a measure of social anxiety^41^. Cronbach’s α for S-SIAS in the current study was 0.94.

#### Depression

Depression symptoms were measured using the Centre for Epidemiological Studies-Depression (CES-D) scale^44^. The CES-D consists of 20 statements which assess individuals' experience of depressive symptoms on a 0 (*rare or none of the time*) to 3 (*Most or all of the time*) Likert-type scale over a 1-week period^44^. Composite scores range from 0 to 60, with higher scores indicating higher experience of depression symptoms. The CES-D has demonstrated excellent internal reliability^44^. Cronbach’s α for CES-D in the present study was 0.94.

### Physiological tests for CVH/CVD indicators

#### BMI

BMI was measured with the Tanita scale which uses Bioelectrical Impedance Technology to determine body composition. BMI values were calculated using the participants' weight and height measurements and reported in kg/m^2^. Higher BMI is associated with poorer CVH^45^.

#### Blood pressure

Systolic and diastolic blood pressure was measured using a calibrated automated sphygmomanometer (Omron sphygmometer model HEM-7121). Prior to measurement, participants were asked to remove any restrictive clothing which could affect their blood flow. They were also asked to refrain from talking and using their mobile phones during testing. Participants were in an upright seated position and, after 5 min rest, three consecutive readings were taken from the right arm over a five-minute period. If large differences in readings were observed, additional measurements were taken. Mean systolic and diastolic blood pressure values in mmHg were calculated from the readings. Higher systolic and diastolic blood pressure are indicators of poor CVH and a risk factor for CVDs^45^.

#### Vascular function

##### Endothelial function

Endothelial function was measured non-invasively using a pulse amplitude tonometry device—EndoPat (2000 Itamar). Use of EndoPat to measure endothelial function has been well validated against industry standard—flow-mediated dilation method^46,47^. Endothelial dysfunction is a reliable predictor of CVH issues, and CVD. In fact, endothelial dysfunction has been identified as the earliest precursor for atherosclerosis (type of CVD)^48,49^. Prior to testing, participants were instructed to lie in a supine position on the laboratory room bed. A deflated blood pressure cuff was placed on the left arm of the participants and two EndoPat finger probes were placed on the tip of each index finger (or middle finger if more suitable) and then inflated. The test took 15 min to complete, and the participants were instructed to stay still and to be quiet for the duration of the test. Baseline or pre-occlusion measurements were recorded for five minutes, followed by five-minute occlusion of the left arm. Occlusion was achieved by inflating the cuff on the participants' upper arm to supra-systolic pressure (60 mmHg above participant’s systolic pressure or to a maximum of 200 mmHg). Occlusion was released after five minutes to induce reactive hyperemia (i.e., interruption of blood flow). Post-occlusion measurements were taken for five minutes. Reactive Hyperemia Index (RHI) was used as an indicator for vascular function. RHI is calculated by dividing the pre-to-post occlusion signal amplitude index in the occluded arm to the corresponding ratio in the control arm to calculate RHI. Lower RHI value is indicative of endothelial dysfunction and poor CVH^50,51^.

##### Arterial stiffness

Arterial stiffness was assessed using a SphygmoCor XCEL AtCor Medical device which utilises central arterial pressure waveform analysis (pulse wave analysis) and carotid-femoral pulse wave velocity as measures of arterial stiffness. Arterial stiffness is a well-established predictor for CVD^52^.

At the start of the test, the participant was asked to lie in a supine position and to minimise movement and speaking during the test. For pulse wave analysis, a brachial cuff was placed on the participants' upper right arm to capture brachial systolic and diastolic blood pressure, and brachial waveform. Results for pulse wave analysis were automatically calculated by SphygmoCor XCEL software. Repeated measurements (minimum two) were taken to ensure consistency. This test took five minutes to complete. Pulse wave analysis results were reported as Augmentation Index (Alx) in this study. Alx is widely accepted as a surrogate measure for arterial stiffness and increased or higher Alx value is indicative of poor CVH^53,54^.

For pulse wave velocity, a cuff was placed around the participants' femoral artery to capture the femoral waveform, and a tonometer was placed on the carotid artery to capture the carotid waveform. In this study, pulse wave velocity results were reported using Pulse Transit Time (PTT) in milliseconds and Carotid-femoral Pulse Wave Velocity (cfPWV) in m/s. The distance between the carotid and femoral arteries was measured, and the velocity automatically determined by dividing the distance by the PTT. Shorter or lower PTT and higher or faster cfPWV suggest arterial stiffness, and potential vascular damage^55^. Both PTT and cfPWV are well established indicators for arterial stiffness^55–57^.

#### Autonomic function

##### Sudomotor function

Sudomotor function was assessed using SUDOSCAN (Impeto Medical). It is a quick and non-invasive test which can detect abnormality in autonomic neuropathy or dysfunction as well as predict diabetes development^58,59^. SUDOSCAN measures sweat chloride concentrations (i.e., sweat gland function) through electrochemical skin conductance (ESC) of hands and feet using reverse ionphoresis. Participants placed their hands and feet on two low voltage (≤ 4 V) stainless steel electrodes for approximately two minutes. Direct current is applied to the electrodes, stimulating the sweat glands to release electrically charged chloride ions onto the skin's surface. Sudomotor function indicators used in the present study are mean feet ESC (µS) and mean hands ESC (µS). Vinik et al. demonstrated the efficacy of SUDOSCAN in measuring hands and feet ESC as indicators of sudomotor function. Lower feet and hands ESC (µS) are indicative of poorer sudomotor function^60^.

##### HRV

HRV was measured using beat-to-beat blood pressure and heart rate readings. Participants were in supine position; readings were captured over a 5-min period using three electrocardiogram leads and PowerLab—a physiological data acquisition device. HRV indicators were calculated in LabChart—a physiological data analysis software. For brevity, we have only included two HRV indicators in the present study—% low frequency power (%LF) and % high frequency power (%HF). Both LF power and HF power are commonly used and well-established indicators of HRV; increase in %LF value and decrease in %HF value are indicative of poor CVH^61,62^.

### Data analytic approach

Both the first research aim—investigate the relationship between loneliness and CVH indicator in young adults, after accounting for social anxiety and depression, and the second study aim—examining differences in the pathways through which loneliness, social anxiety, and depression affect CVH indicators in young adults were answered using Hierarchical Multiple Regression. For research aim 1 –in the hierarchical multiple regression model, the predictor variable was loneliness and outcome variables were CVH indicators. Covariates included age, gender, social anxiety, and depression scores. In Step 1 of the model, age and gender were entered as covariates, followed by the inclusion of social anxiety and depression scores (as covariates—guided by previous empirical research^30,37–39^) in Step 2. Finally, loneliness scores were added in Step 3. This process was repeated for each outcome variable: BMI, blood pressure, vascular function (i.e., endothelial function and arterial stiffness), and autonomic function (sudomotor function and HRV). The findings from this model are presented in Table 4, Model 1.

To ascertain how loneliness, social anxiety, and depression differed in their associations with different CVH indicators, we ran additional two hierarchical multiple regression models. In the first additional model, we examined the unique relationship between social anxiety scores and various CVH indicators in young adults, while controlling for age, gender, depression, and loneliness scores. The model involved three steps. In Step 1, we entered age and gender as covariates. For Step 2, loneliness and depression scores were included as covariates, followed by the addition of the predictor variable—social anxiety—in Step 3. This model was repeated for each outcome variable: BMI, blood pressure, vascular function (i.e., endothelial function and arterial stiffness), and autonomic function (sudomotor function and HRV). The results for this model are showcased in Table 4, Model 2. Likewise, in the second additional model, the unique relationship between depression scores and different CVH indicators in young adults was assessed (after controlling for age, gender, social anxiety, and loneliness scores). The model followed the same three-step process, with age and gender entered in Step 1, loneliness and social anxiety scores (covariates) in Step 2, and depression scores as the predictor variable in Step 3. This model was also applied to each outcome variable, with results presented in Table 4, Model 3.

In addition to the hierarchical multiple regression models, we also ran a correlation matrix to further showcase the interrelated nature of loneliness, social anxiety, and depression. The results for these are presented in Table 3.

## Results

Participant characteristics are presented in Table 1. For categorical variables, percentages were included, and for continuous variables, mean and standard deviation (*SD*) were included. Majority of the participants were single or non-married females who resided with at least one other person and reported no previous diagnosis of CVD.

**Table 1:** Participant characteristics (*N* = 66).

	Percentage/mean (*SD*)
Age (years)	22.70 (4.50)
Gender	
Female	75.8
Male	24.2
Marital status	
Single/never married	72.7
Married/domestic/defacto relationship	27.3
Ethnicity	
Asian Australian or Asian (includes Indian, Indian Australian)	40.0
White (includes Caucasian, European Australian)	33.8
Multiple board categories/others	26.2
Employment status	
Working part time/student/not working	84.8
Working full time	15.2
Level of education completed	
Secondary school	53.0
Vocational education/undergraduate/postgraduate degree	47.0
Living status	
Living with at least one other person	88.5
Living alone	11.5
Chronic CVD conditions	
No CVD present	100.0
At least have one CVD	0.0

The data were analysed using IBM Statistical Package for Social Sciences Version 27.0. The descriptive statistics including, mean and SD values for loneliness, social anxiety, depression, and CVH indicators are presented in Table 2. We have also included the score range observed in the study for aforementioned questionaries and CVH tests in Table 2. All data cleaning, screening, and assumption testing details are included in the Supplementary Material.

**Table 2:** Descriptive statistics (*N* = 66).

	Mean	SD	Score range
Test			
Loneliness	52.75	1.85	28–75
Social anxiety	34.78	2.68	2–59
Depression	24.41	2.45	3–50
BMI			
BMI (kg/m^2^)	24.95	5.47	17.6–44.7
Blood pressure			
Systolic blood pressure (mmHg)	113.21	12.41	90–146
Diastolic blood pressure (mmHg)	72.14	8.91	58–104
Vascular function			
Endopat—RHI	2.12	0.66	1.2–4.0
Pulse wave analysis—Alx	8.26	12.31	-17.0–33.0
Pulse wave velocity—PTT (ms)	114.80	35.01	65–233
Pulse wave velocity—cfPWV (m/s)	5.36	1.64	2.30–12.4
Autonomic function			
Sudomotor—Feet ESC (µS)	82.59	7.31	54–92
Sudomotor—Hands ESC (µS)	74.18	11.91	45–92
HRV—LF (%)	24.74	9.97	6.5–58.1
HRV—HF (%)	42.97	17.27	12.7–80.3

Correlation matrix for loneliness, social anxiety, and depression are presented in Table 3. There were strong, positive, and significant relationships between loneliness, social anxiety, and depression. These directions were as expected.

**Table 3:** Correlational matrix for loneliness, social anxiety, and depression scores.

	1	2	3
1. Loneliness	–	0.74**	0.75**
2. Social anxiety	–	–	0.69**
3. Depression	–	–	–

*N* = 66. Significant *p-*values are bolded, **p < 0.001.

The hierarchal multiple regressions results are presented below in Table 4. As mentioned previously, Model 1 includes the results for study aim 1—the relationship between loneliness and CVH indicators in young adults after accounting for social anxiety and depression. The results revealed that after controlling for age, gender, social anxiety, and depression, higher loneliness was significantly associated with lower or shorter PTT (i.e., measure for arterial stiffness) in young adults. Additionally, the *R*^*2*^_*change*_ statistic also shows that loneliness scores explain 15% of the unique variance in PTT among young adults.

**Table 4:** Hierarchal multiple regression models.

Outcome variables	Model 1—loneliness (predictor)			Model 2—social anxiety (predictor)			Model 3—depression (predictor)		
	β	*R*^*2*^_*change*_	*p*	β	*R*^*2*^_*change*_	*p*	β	*R*^*2*^_*change*_	*p*
BMI									
BMI (kg/m^2^)	− 0.14	0.006	0.54	0.20	0.15	0.31	0.19	0.014	0.33
Blood pressure									
Systolic blood pressure (mmHg)	0.10	0.000	0.93	− 0.21	0.017	0.27	0.15	0.009	0.42
Diastolic blood pressure (mmHg)	− 0.083	0.002	0.71	0.068	0.002	0.74	0.11	0.005	0.57
Vascular function									
Endopat—RHI	0.30	0.027	0.20	0.21	0.16	0.32	− 0.43	0.073	**0.036**
Pulse wave analysis—Alx	0.026	0.000	0.90	0.11	0.004	0.57	− 0.037	0.001	0.84
Pulse wave velocity—PTT (ms)	− 0.70	0.15	**0.002**	0.54	0.10	**0.008**	0.238	0.023	0.20
Pulse wave velocity—cfPWV (m/s)	0.28	0.024	0.20	− 0.24	0.02	0.24	− 0.035	0.000	0.85
Autonomic function									
Sudomotor—Feet ESC (µS)	− 0.18	0.01	0.38	0.33	0.04	0.09	0.17	0.011	0.36
Sudomotor—Hands ESC (µS)	0.16	0.008	0.45	0.16	0.008	0.45	− 0.012	0.000	0.95
HRV—LF (%)	− 0.40	0.045	0.068	0.092	0.003	0.63	0.22	0.19	0.23
HRV—HF (%)	0.19	0.011	0.39	− 0.38	0.053	0.06	0.089	0.003	0.65

*N* = 66. Hierarchical Multiple Regression analyses results. β = Standardised regression parameter estimates. β values specified in the table are for the last predictor variable entered in each model (italicised here). Predictors entered: Model 1—Age, gender, depression, social anxiety, *loneliness.* Model 2—Age, gender, loneliness, depression, *social anxiety*; Model 3—Age, gender, loneliness, social anxiety, *depression.*

Significant values are bolded, *p* < 0.05.

Results for two additional hierarchal multiple regressions assessing study aim two (investigating whether loneliness, social anxiety, and depressions differ in their associations with different CVH indicators in young adults) are presented in Table 4—Models 2 and 3. Higher social anxiety was associated with higher PTT (Table 4, Model 2), after controlling for covariates. Lastly, we found higher scores for depression to be significantly associated with lower RHI (i.e., measure of endothelial function) among young adults, after controlling for age, gender, loneliness, and social anxiety (Table 4, Model 3). Depression scores also accounted for 7.3% of the variance in RHI scores.

## Discussion

The aim of the present study was to investigate the unique relationship between loneliness and CVH indicators in young adults after controlling for confounding effects of depression and social anxiety. We also examined how loneliness, social anxiety, and depression differ in their associations with various CVH indicators in young adults.

We found a unique and significant relationship between loneliness and one of the vascular function indicators in young people. Higher loneliness in young adults was associated with lower PTT, after controlling for social anxiety and depression (Model 1 in Table 4). Shortened PTT is an early sign of arterial stiffness and vascular dysfunction^55^. A lower PTT may occur due to a sudden increase in blood pressure, leading to a rise in vascular tone and subsequent arterial wall stiffness^57,63^. The results of this study make a unique contribution to the literature by demonstrating that young adults with loneliness may already be exhibiting signs of vascular dysfunction or damage, evident as early as in their twenties (mean age for the present study was 22.70 years). These findings are alarming as they indicate that young adults experiencing higher levels of loneliness may also have shorter PTT, potentially contributing to arterial stiffness and reducing the arteries' ability to adapt to changes in blood pressure and flow. Consequently, prolonged arterial stiffness and vascular dysfunction may result in arterial damage and increase their risk of developing CVDs^64–66^.

Additionally, some differences in the pathways through which loneliness, depression, and social anxiety influence vascular function in young adults were noted, mainly between loneliness and depression. Higher depression was significantly associated with lower RHI—an indicator for endothelial dysfunction, even after controlling for loneliness and social anxiety (Table 4 Model 3). The endothelium is a membrane surrounding the insides of the heart and blood vessels and its main role is to maintain homeostasis by regulating vasodilation and vasoconstriction of the blood vessels^67^. Arterial stiffness, on the other hand, refers to the rigidity or stiffness of the arterial wall^52^. Both endothelial dysfunction and arterial stiffness are related to vascular damage/dysfunction and have been identified as independent risk factors for several CVDs^48,52,65^. Indeed, endothelial dysfunction is widely acknowledged as one of the earliest indicators of atherosclerosis (i.e., type of major CVD)^48,49^, although it can potentially be reversible in early stages^68^. On the other hand, arterial stiffness is a more progressed form of vascular damage and a contributing factor to a number of other CVDs, such as myocardial infarction, heart failure, and even mortality^66^. It is possible that CVH abnormalities such as endothelial dysfunction manifest earlier in young people with depression symptoms. Indeed, several studies have found an association between depression and endothelial dysfunction^69,70^. However, it should be noted that we did not control for any other known covariates of depression (e.g., genetics, previous diagnosis of major depressive disorder). These findings indicate that young people with more depressive symptoms are more likely to present with earlier indicators for poor CVH, whereas young people who report higher loneliness may present equally concerning impairment in CVH, although in a more progressed form.

While previous research suggests that loneliness may be an antecedent to depression^30,37^, it is possible that the relationship between these constructs and how they affect CVH may be more complex and closely intertwined than we anticipated (e.g., reciprocal or cyclic in nature). Interestingly, in the current study, higher social anxiety was associated with higher PTT in young adults (after controlling for loneliness and depression, Table 4 Model 2). In other words, higher social anxiety was related to improved vascular function. This finding was unexpected, as we would assume social anxiety to follow the same trajectory and directionality as loneliness in relation to PTT, given that previous research indicates a reciprocal relationship between the two constructs^38,39^. Similar to depression, it should be noted that we did not account for the influence of any confounding variables for social anxiety (e.g., previous social anxiety disorder diagnosis) which may have affected the results. Future research with a broader consideration of the confounding variables could help validate and extent these findings. Nonetheless, ultimately, both higher loneliness and depression are related to impaired vascular function and poor CVH in young adults. Future research focusing on loneliness and depression, and how they impact CVH and increase risk for CVD, especially in young adults, would be a pertinent and strategic approach to facilitate early intervention and disease prevention.

We found no relationship between loneliness and autonomic function, BMI, and blood pressure in young adults after controlling for social anxiety and depression (Model 1 in Table 4). While these results are inconsistent with some of the reviewed studies^28–32^, they are congruent with some other research findings^33–36^. There are several possible explanations for the non-significant findings. For instance, hardly any of the abovementioned studies with inconsistent results accounted for the influence of concomitant mental health issues such as social anxiety and depression. Thus, it is plausible that some of the predictive and explanatory power attributed to loneliness in regard to CVH indicators in previous research was in fact the influence of concurrent mental health symptoms, such as social anxiety and depression. Indeed, findings from some of the congruent studies demonstrate that a positive relationship between BMI and loneliness is only present when relevant covariates are not considered^35,36^. Our findings further emphasise the importance of controlling for highly correlated mental health symptoms, such as social anxiety and depression, when investigating the influence of loneliness on health or CVH, especially in young adults.

Additionally, it is also possible that some of the CVH indicators used in previous studies were not sufficiently sensitive. Most research on loneliness and CVD has utilised CVH indicators such as blood pressure, HRV, and BMI. It may be that these CVH indicators are not sensitive or discernible enough to detect small differences or associations concerning loneliness and its influence on CVH, particularly in otherwise healthy young adults with no existing CVD issues, as was the case in the present study. These findings further highlight the necessity of using early or subclinical markers for CVD/CVH such as vascular function, especially when working with young adults, as they are unlikely to present with significant CVD/CVH issues. These recommendations are also supported by our results for loneliness and PTT. As mentioned previously, PTT refers to the time it takes for a pulse wave to travel between two arterial sites; the speed at which this arterial pulse wave travel’s is inversely proportional to blood pressure^71,72^. PTT is understood to be strongly influenced by blood pressure^71–75^. However, the pulse wave, from which PTT is calculated, moves much faster than blood. Consequently, pulse wave and PTT can detect minute and subtle physiological or cardiovascular changes that may not be discernible when using more common CVH such as direct blood pressure measurement. Our findings suggest that PTT, which is a subclinical indicator for CVH/CVD, may be a better predictor for impaired vascular function and poor CVH than more traditionally used measures such as blood pressure, HRV, and BMI, especially in research involving young adults. Future research should focus on the use of subclinical CVH indicators as they may help predict CVH issues earlier in life^52^ and as such, facilitate primary and secondary prevention of cardiovascular events. An alternate explanation for the observed lack of relationship between loneliness and some of the CVH indicators (e.g., blood pressure) could be due to the diverse roles these factors play in influencing CVH outcomes. Rather than directly influencing CVH, it is possible that some of the CVH indicators may have a moderating or mediating effect on the relationship between loneliness and CVH markers.

The present study has some limitations. It is important to consider the cross-sectional study design, small sample size, generalisability, and ecological validity of our findings. For example, our participants were primarily female university students who spoke English as a first language, resided in the state of Victoria, and who were living with other people. As such, our sample may not be representative of all Australian young adults. Additionally, use of cross-sectional study design restricted our ability to draw causal inferences or ascertain directionality between loneliness and various CVH indicators in young adults. Future researchers should adopt a longitudinal study design to potentially replicate and extend the findings from the current study and establish causality. For instance, future research could focus on investigating the influence of other common covariates related to CVDs (e.g., genetics and medical history). Other methodological and statistical limitations include the different time periods over which loneliness and depression symptoms were measured (i.e., loneliness was assessed over the past month using UCLA-LS, while depression was assessed over the past seven days using CES-D). Furthermore, it is important to note that no additional corrections for multiple comparisons were applied to the data, as hierarchical multiple regression was utilised. In this approach, covariates and predictors were entered into the model in a stepwise manner (see Table 4), and the study was not exploratory in nature, as only one a-priori hypothesis was tested at a given time^76^. Nonetheless, these methodological and statistical shortcomings should be taken into account when interpreting the results. Additionally, we do not know how different levels of loneliness, depression, and social anxiety (e.g., low loneliness and high depression) interact to influence different CVH markers in young adults over time. Investigating these relationships will help establish causality and better elucidate the multifaceted relationship between loneliness, depression, and social anxiety, and their combined and individual effects on CVH in young adults.

### Clinical implications

While we were limited by our ability to generalise the results to a broader young adult population and establish causality, we believe our findings have important clinical implications, particularly in delivering early intervention for CVD. Our findings suggest that young adults with loneliness are at a higher risk for developing CVD. As such, we would urge healthcare professionals to measure loneliness when assessing a patient’s risk of developing CVD. Doing so would allow healthcare professionals to develop more personalised and holistic interventions, focusing on many of the interdependent factors that may contribute to the relationship between higher loneliness and poorer CVH in young adults, as well as primary and secondary CVD prevention. The clinical implications and applications of our findings are more grounded in preventative healthcare, rather than the more traditional approach of curative healthcare^77^. Long term studies are urgently needed to clearly establish the link between loneliness and risk of developing CVD in young adults, after accounting for the role of various highly correlated mental health issues. In particular, how does more persistent states of loneliness (i.e., chronicity) influence the development of CVD?

## Conclusion

We demonstrated higher loneliness is associated with poorer CVH, in particular impaired vascular function (i.e., a well-established risk factor for several CVDs) in young adults, after controlling for the influence of known confounding mental health issues. Additionally, we also highlight the different pathways through which loneliness and depression may affect CVH in young adults, namely arterial stiffness and endothelial function respectively.

### Supplementary Information

Supplementary Information.

## Data Availability

The datasets generated and/or analysed during the current study are not publicly available due ethical restrictions, but are available from the corresponding author on reasonable request.
