# Supplementary material for: Investigating the relationship between early cardiovascular disease markers and loneliness in young adults
Source: Sci Rep. 2024 Jun 20;14:14221. doi: 10.1038/s41598-024-65039-8 (PMC11190220; doi:10.1038/s41598-024-65039-8)
Supplement: Supplementary file 1 — Supplementary Information. [file 41598_2024_65039_MOESM1_ESM.docx]

**Supplementary Material**

**Data Cleaning, Screening, and Assumption testing**

Hierarchical multiple regression were the main analyses used in the current study. All assumption testing for multiple regression analyses were satisfactory. Normality was assessed by visual inspection of histograms and analysing absolute and z-scores values for skewness and kurtosis for all predictors, covariates, and outcome variables. There were no significant deviations from normality in line with Hair et al. [1]. There were no substantial outliers, univariate (assessed using z-scores of ±3.29, at *p* < .001) or multivariate (assessed using Mahalanobis distance, χ^2^ value larger than ±20.51 (i.e., the critical χ^2^ value for *df* = 5 at *p* < .001)). There was no evidence of multicollinearity and non-independence of errors (tolerance value > 0.1 and VIF < 10; Durbin Watson statistic between 1.5 and 2.5 for all outcome/dependent variables) [2].

**References**

1. Hair, J. F., Black, W. C., Babin, B. J., & Anderson, R. E. *Multivariate data analysis*. (Pearson Higher Education, 2013).
2. Allen, P., Bennett, K., & Heritage, B. *SPSS statistics version 22: A practical guide*. (Cengage Learning Australia, 2014).
